# Case Report: Dacomitinib Overcomes Osimertinib Resistance in NSCLC Patient Harboring L718Q Mutation: A Case Report

**DOI:** 10.3389/fonc.2021.760097

**Published:** 2021-12-02

**Authors:** Qian Shen, Jingjing Qu, Zhen Chen, Jianying Zhou

**Affiliations:** ^1^ Department of Respiratory Disease, Thoracic Disease Centre, The First Affiliated Hospital, College of Medicine, Zhejiang University, Hangzhou, China; ^2^ Laboratory Medicine and Pathology, The First Affiliated Hospital, College of Medicine, Zhejiang University, Hangzhou, China

**Keywords:** NSCLC, osimertinib, EGFR L718Q mutation, dacomitinib, brain metastatis

## Abstract

**Background:**

Advanced non-small cell lung cancer (NSCLC) harboring epidermal growth factor receptor (EGFR) mutations has been successfully treated with tyrosine kinase inhibitors (TKIs). However, resistance to osimertinib, a third-generation TKI, can be difficult to overcome in this small subset of patients and is attributed to secondary resistant mutations. Here, we report a case of acquired EGFR L858R/L718Q mutation with advanced NSCLC that resistant to osimertinib, which was successfully overcome using dacomitinib.

**Case Presentation:**

A 64-year-old non-smoker woman was diagnosed with stage IV non-small cell lung adenocarcinoma with EGFR L858R mutation and brain metastasis in November 2018. Treatment with gefitinib and gamma knife radiosurgery was started as the first-line treatment. After 7 months, she experienced disease progression with increased primary lung lesions and switched to osimertinib based on an acquired EGFR T790M mutation. After another 4 months, the disease progressed, and she was switched to chemotherapy. During chemotherapy, brain MRI showed an increasing number of parietal lobe metastases. Hence, gamma knife radiosurgery was performed again. After 12 months, the disease progression resumed, and an EGFR L718Q mutation was found on biopsy. The patient was then challenged with dacomitinib, and the disease was partially responsive and under control for 6 months.

**Conclusion:**

Currently, there are no established guidelines for overcoming osimertinib resistance caused by the L718Q mutation. The acquired EGFR L718Q mutation in subsequent resistance to osimertinib could be overcome using dacomitinib, indicating a promising treatment option in the clinic.

## Introduction

In China, approximately 50% of all non-small cell lung cancers (NSCLCs) have an epidermal growth factor receptor (EGFR) mutation (1). It is well known that advanced NSCLC harboring EGFR mutations has been successfully treated with tyrosine kinase inhibitors (TKIs). Unfortunately, although uncommon, resistance to the third-generation TKI osimertinib can be difficult to overcome in a small subset of patients and has been attributed to secondary resistant mutations. The EGFR L718Q mutation, identified in 8% of Chinese osimertinib-resistant NSCLC patients, has been found to independently lead to osimertinib resistance by stabilizing its non-reactive conformation, but an effective treatment directed at this rare mutation has yet to be identified (1). Several reports have shown that second-generation EGFR TKIs can overcome osimertinib resistance due to concomitant EGFR L858R/L718Q mutations (1, 2).

## Case Presentation

**Figure 1:**
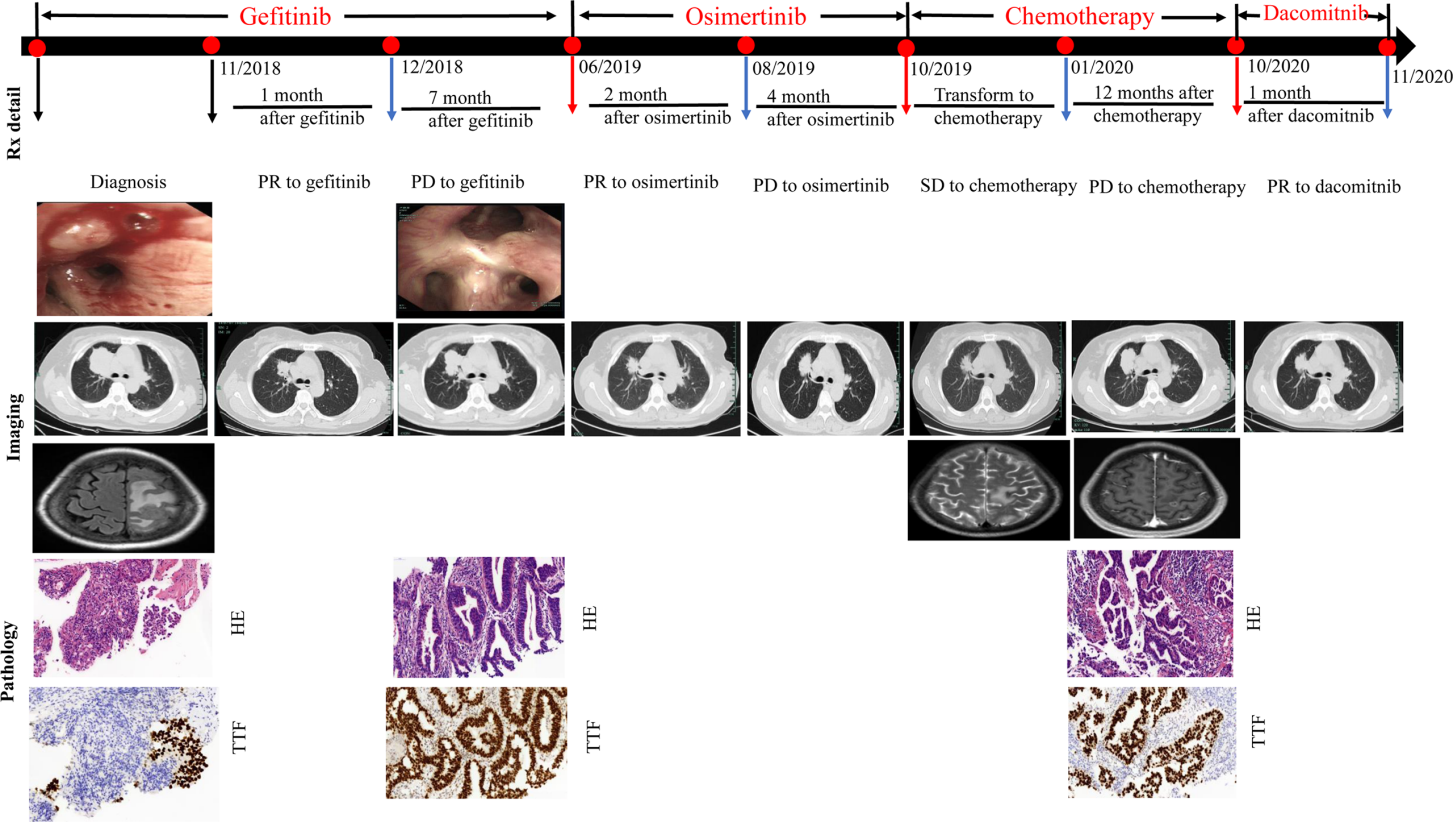
Evolution of the disease in case presentation. CT, computed tomography; HE, hematoxylin and eosin; Syn, synaptophysin; Rx, treatment.

A 64-year old non-smoker female patient with a 15-year work history at an asbestos factory visited our hospital with complaints of dizziness and difficulty ambulating the right leg in November 2018. Bronchoscopy revealed a mass in the upper lobe of the right lung. Chest computed tomography (CT) showed a large space-occupying lesion measuring 5.7x4.6 cm with mediastinal lymph node enlargement. Magnetic resonance imaging (MRI) showed multiple lesions in both cerebral hemispheres, with the larger lesion sized 2.3x2.1 cm located in the left parietal lobe (**Figure 1**). The level of carcinoembryonic antigen(CEA) was measured to be 61.4 ng/mL in this patient. Immunohistochemistry of the right upper lobe mass biopsy revealed lung adenocarcinoma: Ki-67 (+, 80%), thyroid transcription factor-1 (TTF-1) (+), and napsin A (+). Amplification refractory mutation system (ARMS) testing revealed an EGFR L858R mutation. Finally, the patient was diagnosed with clinical stage IV (T3N2M1b) non-small-cell lung adenocarcinoma with brain metastasis. The patient was treated with gefitinib and gamma knife radiosurgery because the primary lung adenocarcinoma sample harbored EGFR exon 21 L858R mutation.

After four weeks (in December 2018), chest CT showed that the space-occupying lesions in the right upper lobe of the lung were significantly decreased in size compared to the previous scan in November 2018. The patient experienced a partial response (PR) according to the Response Evaluation Criteria in Solid Tumors (RECIST) 1.1. However, progressive disease was then identified with the primary lesion of the right upper lung becoming enlarged until June 2019, yielding a progression-free survival (PFS) of 7 months. At this time, right lung rebiopsy was performed for pathological detection and genetic testing. Immunohistochemical examination in June 2019 revealed lung adenocarcinoma: TTF-1 (+) and napsin A (+). ARMS testing revealed the T790M and L858R mutations. The patient was treated with osimertinib in June 2019. Follow-up in August 2019 showed regression of the primary lung lesions, but had increased in size again by October 2019. As a result, chemotherapy with pemetrexed, and carboplatin was initiated in October 2019 as a third-line treatment. In January 2020, the patient was admitted to the hospital because of severe headache, dizziness, and nausea. Brain MRI in January 2020 showed an increasing number of parietal lobe metastases. Gamma knife radiosurgery was repeated in January 2020, and pemetrexed chemotherapy was continued with the addition of bevacizumab as an anti-angiogenic agent.

**Figure 2:**
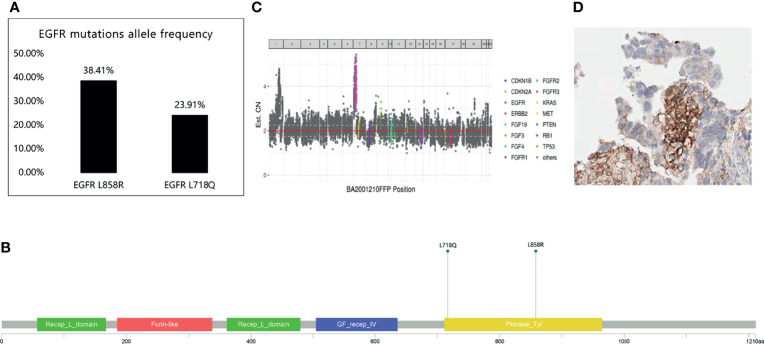
520 genes showed EGFR L858R and L718Q mutations. **(A)** EGFR mutations allele frequency. **(B, C)** The location of EGFR L858R and EGFR L718Q mutation. **(D)** The expression of programmed death-ligand 1 (PD-L1).

In June 2020, chest CT showed a slight regression in the size of both lung masses. Therefore, chemotherapy with pemetrexed and bevacizumab was continued. Brain MRI in October 2020 showed no significant enhancement in the brain parenchyma. Because the primary lung lesion continued to grow in size, a rebiopsy of the right upper lobe lesion was performed in October 2020. Immunohistochemical analysis revealed lung adenocarcinoma: TTF-1 (+) and napsin A (+). Detection of 520 genes showed EGFR L858R and L718Q mutations and a programmed death-ligand 1(PD-L1) expression level of 20%(**Figure 2**). The L718Q mutation is considered to be one of the underlying reasons for osimertinib resistance and disease progression. In October 2020, after all the exploring therapeutics failed, second-generation TKI dacomitinib was administered (45 mg/day orally). The patient tolerated the treatment well with grade 1 diarrhea. The diameter of the primary lung lesion in the right upper lobe decreased from 53 to 39 mm in November 2020, indicating a partial response. The patient again experienced progressive disease five months later, and the primary lung lesion increased in size.

## Discussion and Conclusion

Our patient was diagnosed with a primary lung adenocarcinoma harboring the primary EGFR L858R mutation. Due to disease progression, first-line gefitinib was switched to osimertinib. Gefitinib resistance was attributed to a secondary T790M mutation found on rebiopsy. After the switch, further progression of the lung lesions was observed within four months. The resistance to the second-line osimertinib therapy was attributed to the rare EGFR L718Q mutation found on rebiopsy. Several reports have shown that an effective strategy to overcome osimertinib resistance resulting from L718Q mutation was to use second-generation EGFR TKIs (3, 4). Therefore, we changed the therapy to dacomitinib. Among the candidate TKIs, dacomitinib was chosen over afatinib because it has been speculated that dacomitinib might be active in the brain due to its increased brain penetration compared to afatinib (5).

In the clinic, no experience of nervous system (CNS) failure to osimertinib has been reported. Chemotherapy, combined with whole-brain radiation, may be an option, but it is not suitable for most patients with leptomeningeal metastasis with poor performance status. Bevacizumab has been reported to be effective in reducing malignant effusion and edema to relieve symptoms (6, 7). Based on these reports, the patient in the present study was challenged with bevacizumab; however, she did not respond to this drug.

Using the data from patients with NSCLC, cell lines, and computer models, Jacquulyne Robichaux et al. found that EGFR mutations can be divided into four functional subgroups including classcical-like mutation, T790M-like mutations, exon 20 insertions and pocket volume reducing (PVR) mutation (interior of the ATP binding pocket or in α-helix/P-loop) (8). The mechanisms of resistance to third-generation EGFR TKIs have been demonstrated in recent years. The most common acquired mutations occur in codon 797 (i.e., C797S), while other rare mutations at positions L718, L792, or G719 have also been reported recently (9–11). The EGFR L718Q mutation, which accounts for 7.3% to 9.7% of osimertinib resistance, is located in the P-loop within the ATP binding site of the EGFR kinase domain (10). A crystallographic model demonstrated that L718Q mutation can cause steric hindrance, thus reducing the binding affinity of osimertinib. *In vitro* studies have demonstrated that cells with EGFR L718Q alone may respond to first- and second-generation EGFR TKIs. However, cells harboring both EGFR L858R and L718Q variants were resistant to osimertinib and gefitinib but were sensitive to second-generation EGFR TKIs afatinib or dacomitinib when T790M was lost (12).

Understanding and identifying resistance mechanisms is imperative because it allows us to strategize a patient-specific treatment regimen that can be adjusted to provide optimal response. Several resistance mechanisms leading to the use of EGFR TKIs have been elucidated in recent years. The L718Q mutation has been shown to confer the highest osimertinib resistance by interfering with the alanine ring of osimertinib, subsequently causing steric hindrance and leading to drug resistance. Currently, there are no established guidelines for overcoming osimertinib resistance caused by the L718Q mutation. For NSCLC patients with osimertinib resistance caused by tertiary EGFR mutations, further investigations are needed to elucidate the resistance mechanisms that can help direct the development of new treatment strategies.

## Data Availability Statement

The raw data supporting the conclusions of this article will be made available by the authors, without undue reservation.

## Ethics Statement

The studies involving human participants were reviewed and approved by First Affiliated Hospital, College of Medicine, Zhejiang University. The patients/participants provided their written informed consent to participate in this study. Written informed consent was obtained from the individual(s) for the publication of any potentially identifiable images or data included in this article.

## Author Contributions

QS and JZ provided patient information. JQ collected the data and wrote the manuscript. JQ critically revised the manuscript for intellectual content. ZC provided pathological photos. QS and JZ were responsible for the study conception, design, and acquisition of financial support. All authors contributed to the article and approved the submitted version.

## Funding

This work was supported by the Medicine and Health Project of Zhejiang Province, China (grant number 2018KY061).

## Conflict of Interest

The authors declare that the research was conducted in the absence of any commercial or financial relationships that could be construed as a potential conflict of interest.

## Publisher’s Note

All claims expressed in this article are solely those of the authors and do not necessarily represent those of their affiliated organizations, or those of the publisher, the editors and the reviewers. Any product that may be evaluated in this article, or claim that may be made by its manufacturer, is not guaranteed or endorsed by the publisher.
